# Clinical and Imaging Predictors of Disease Progression in Type B Aortic Intramural Hematomas and Penetrating Aortic Ulcers: A Systematic Review

**DOI:** 10.3390/diagnostics12112727

**Published:** 2022-11-08

**Authors:** Elda Chiara Colacchio, Francesco Squizzato, Michele Piazza, Mirko Menegolo, Franco Grego, Michele Antonello

**Affiliations:** Department of Cardiac, Thoracic, Vascular Sciences and Public Health, Vascular and Endovascular Surgery Section, University of Padova, Azienda Ospedale-Università di Padova, 35128 Padova, Italy

**Keywords:** penetrating aortic ulcer, penetrating atherosclerotic ulcer, intramural aortic hematoma, type B intramural hematoma

## Abstract

Background: This work aims to review recent literature on penetrating aortic ulcers (PAUs) and intramural hematomas (IMHs), in order to identify clinical and imaging factors connected to aortic-related adverse events (AAE). Methods: We performed a systematic review according to the Preferred Reporting Items for Systematic review and Metanalyses (PRISMA) guidelines. An electronic search was conducted on Medline and Embase databases. We included articles reporting on PAUs and/or IMHs localized in the descending thoracic and/or abdominal aorta and analyzing clinical and/or radiological markers of AAE. Results: Of 964 records identified through database searching, 17 were incorporated in the present review, including 193 and 1298 patients with type B PAUs and IMHs, respectively. The 30-days aortic-related mortality (ARM) was 4.3% and 3.9% for PAUs and IMHs. A total of 21% of patients with IMHs underwent intervention during the follow-up period, and 32% experienced an AAE. PAU markers of AAE were minimum depth (ranging from 9.5 to 15 mm) and diameter (≥12.5 mm). Maximum aortic diameter (MAD) cut-off values ranging from 38 to 44.75 mm were related to AAE for IMHs, together with ulcer-like projection (ULP) of the aortic wall. Conclusions: Despite data heterogeneity in the literature, this PAU- and IMH-focused review has highlighted the imaging and clinical markers of disease progression, thus identifying patients that could benefit from an early intervention in order to reduce the AAE rate.

## 1. Introduction

Acute aortic syndromes (AASs) are a wide spectrum of pathologies characterized by various degrees of disruption of the aortic wall integrity. They include aortic dissections (ADs), intramural hematomas (IMHs), penetrating aortic ulcers (PAUs) and traumatic aortic injuries (TAIs). These conditions may coexist in the same patient and/or evolve in another one of the same group [1].

PAUs are focal disruption of the intima extending to the media, most frequently localized in the descending thoracic and abdominal aorta [2], and occurring in patients with severe atherosclerosis and older than those with AD. An IMH is a bleed within the aortic media, without evidence of intimal tears at computed tomography angiography (CTA). PAUs and IMHs have some elements in common, such as the age of appearance and the preferential location; thus, they have traditionally been considered as strictly connected. However, recent literature seems to suggest that they could be different pathologies [3,4,5,6] according to risk factors, clinical behavior and imaging appearance.

Moreover, many radiologic patterns, once identified as PAUs associated to IMH, are now considered as completely different characteristics potentially related to high risk IMH, the so-called ulcer-like projections (ULPs) or focal intimal disruptions (FIDs), defined as areas of focal enhancement within the hematoma, with a clear communication with the true lumen, and not associated with atherosclerotic plaques.

Although AASs represent a potentially life-threatening condition, and prompt medical and/or procedural treatment is considered mandatory [7], the specific clinical course of IMHs and PAUs is still unclear, especially in the long-term. In fact, since their incidence is relatively low [8], specific randomized controlled trials (RCT) are lacking, and even observational reports often analyze overall AASs behavior or do not distinguish between the clinical course of these pathologies localization (ascending or descending aorta, aortic arch). Furthermore, literature often merges acute symptomatic and chronic incidental findings.

In this review, we aimed to analyze recent literature on acute-type B PAUs and IMHs in order to detect clinical and radiological features related to worsened outcome or disease progression requiring intervention, thus identifying cases that could benefit from an early intervention even if not immediately complicated.

## 2. Materials and Methods

### 2.1. Search Strategy

We performed a systematic review according to the Preferred Reporting Items for Systematic review and Metanalyses (PRISMA) guidelines [9]. Electronic research was conducted in August 2022 on Medline and Embase databases, using the terms “penetrating aortic ulcer”, “penetrating atherosclerotic ulcer”, “intramural hematoma”, “intramural aortic hematoma” and “type B intramural hematoma”, without specific terms on management and therapy, since our goal was to identify all articles where the authors analyzed any factors leading to adverse outcomes. The filters applied in this initial selection were language (articles in English), time frame (the last 10 years) and type of articles, excluding guidelines, reviews, systematic reviews and metanalysis.

### 2.2. Study Selection and Eligibility Criteria

**Figure 1:**
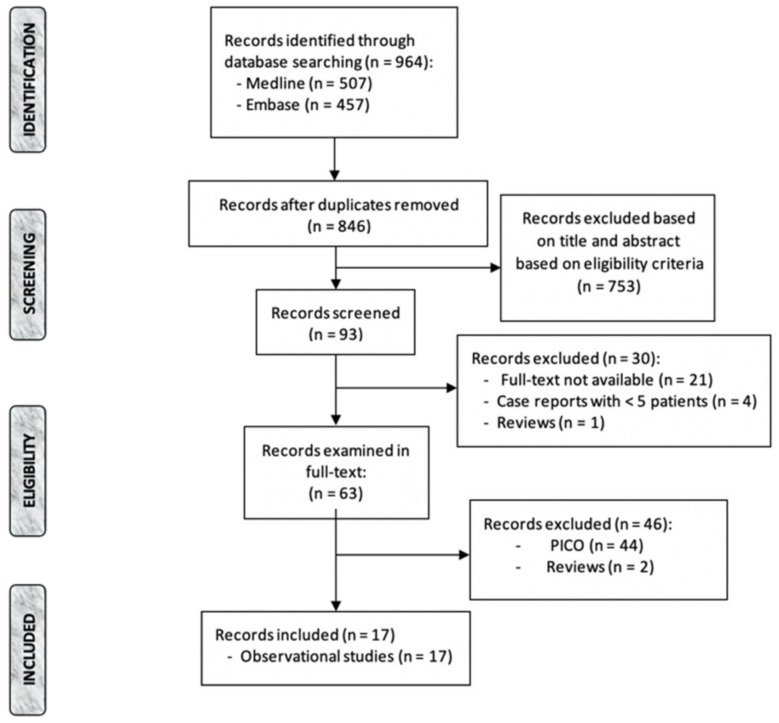
Flow-chart of screening procedure according to the Preferred Reporting Items for Systematic review and Metanalyses (PRISMA) guidelines [9].

Data were imported in a reference management software (Mendeley Desktop 1.19.8, © Mendeley Ltd.) that allowed duplicates’ removal and articles’ initial screening by title and abstract, and finally by full texts (Figure 1). All steps of the selection were independently performed by two researchers (E.C.C. and F.S.). Discrepancies and disagreements were resolved by discussion.

Inclusion criteria were articles reporting PAUs’ and/or IMHs’ management, with disease localization in the descending thoracic or abdominal aorta, and analyzing clinical and/or radiological features linked to adverse aortic-related events (AAE), including aortic-related mortality, disease progression, disease evolution in another AAS and intervention.

In the first selection by title and abstract, we excluded case reports analyzing less than 5 patients, disease only affecting the ascending aorta and aortic arch, articles only reporting on ADs, studies comparing treatment between aortic aneurysms and AASs, expert consensus documents and articles evaluating a single type of stent-graft behavior. We subsequently excluded articles will unavailable full texts.

In the final full-text screening, we excluded articles whose authors only reported their experience without outcome-related factors’ identification.

### 2.3. Data Extraction

The selected baseline information (Table 1 and Table 2) were the following: author; year of publication; overall number of patients; number of PAUs and IMHs; diseases’ localization in the descending thoracic and abdominal aorta (summarized as type B disease according to Stanford classification); acute onset; mean or median follow-up; primary and secondary outcomes selected by authors; clinical, laboratory and radiologic patterns whose association with AAEs was determined.

We also collected data on clinical progression, particularly the number of patients undergoing an intervention (endovascular, surgical or hybrid) in the acute phase and/or follow-up, the number of cases of disease progression, defined according to each author, and AAE rates. We also identified rates of aortic-related death, evolution of measured radiologic patterns, clinical and/or radiologic features significantly associated with disease progression and/or poor outcome (Table 3 and Table 4).

### 2.4. Imaging Evaluation

All studies included patients with baseline and follow-up CTAs, in order to describe the imaging features that could be used as a monitor of disease severity and in order to detect markers of disease progression (DP) and/or poor prognosis.

All articles on PAUs reported ulcer location, number, depth, width, neck width and maximum aortic diameter (MAD). Two authors also described the presence and thickness of periaortic hematoma [10,11].

In IMH reports, the authors measured MAD, maximum hematoma thickness (MHT) and ulcer-like projections (ULPs, also called focal intimal disruptions—FIDs), defined as a focal contrast enhancement within the hematoma, with a clear communication with the aortic lumen and in the absence of atherosclerosis and/or calcified plaques, thus differentiating them from PAUs. Other recorded features were aortic tortuosity and intramural blood pools (IBP), namely focal contrast enhancement in the hematoma without communications with the true lumen [12]. In one article, authors analyzed IMHs via 18F-fluorodeoxyglucose (18F-FDG) positron emission tomography/computed tomography (PET/CT) and described the maximum standardized uptake of the aortic wall (SUV_max_), the maximum standardized uptake of the superior vena cava (SUV_svc_) and the SUV_max_/SUV_svc_ ratio, called target to blood ratio (TBR) [13].

## 3. Results

### 3.1. Study Selection and Overall Characteristics

The records identified through database searching were 964 (PubMed: 507; Embase: 457), and records screened in full-text were 63. Of these, 17 studies were included in the present review, all observational and retrospective (Figure 1). Two studies only reported on PAUs [11,14], two studies reported on PAUs and IMHs [5,10] and thirteen articles only concentrated on IMHs [12,13,15,16,17,18,19,20,21,22,23,24,25].

Overall, of 237 patients with PAUs, in 193 the disease was localized in the descending thoracic and abdominal aorta (type B) (81%); two studies also included PAUs located in the aortic arch [11,14]. In 218 cases (92%), authors analyzed PAUs with an acute onset.

The number of patients with IMHs was 1338, of which 1298 were type B (97%) and 1327 were analyzed from the acute phase (99%).

Follow-up in the studies reporting on PAUs ranged from 23 months [11] to 8.1 years [5], while the minimum and maximum follow-up period in articles also reporting on IMHs were 42 days [13] and 8.1 years [5].

In articles where the type of treatment was specified, the majority of patients underwent endovascular repair.

### 3.2. Outcomes

#### 3.2.1. PAU

Three studies reported 30 days aortic-related mortality (ARM) [10,11,14] (9/209, 4.3%) (Table 1).

Janosi et al. [14] analyzed 63 cases of acute PAUs of which 52.3% (33/63) underwent thoracic endovascular aortic repair (TEVAR) in the first 14 days, and in 30/63 (47.6%) cases the time frame between diagnosis and intervention was 40 ± 39 days; in 21 of all treated cases (33%), patients were asymptomatic, and the indication for treatment was disease progression on imaging, such as increase in PAU size or development of a new IMH. During follow-up, 12 patients (19%) underwent a new intervention because of endo-leaks or aortic disease progression. In-hospital mortality was 7.9%, while survival estimates at 1, 5 and 10 years were 88.4%, 82.2% and 65.7%.

Yang et al. [11] reported a 30 days AAE rate of 28.44%, while this value increased to 31.19% during follow-up. AAE was defined as a composite outcome comprehensive of death, progression to AD and ulcer worsening. The authors noticed an increase in PAUs’ diameter and depth in 23/109 patients (21%).

In their analysis on 28 type B PAUs, Squizzato et al. [5] registered an increase in aortic diameter of 0.4 mm/year (IQR 0–1.8), an increase in PAU depth and width of 0.5 mm/year (IQR 0.1–1.4) and 1.1 mm/year (IQR 0.7–1.7), respectively, and an increase in neck width of 1.1 mm/year (IQR 0.6–4.6). Clinical PAU progression was considered when intervention, aneurysm formation, death or evolution into a different AAS occurred, and freedom from this outcome at 1, 5 and 10 years was 90.5%, 70.6% and 45.8%, respectively, while estimated disease resolution at 1, 5 and 10 years were 0%, 10.6% and 10.6%, respectively.

In the study of Piazza et al. [10], five patients underwent intervention (four endovascular and one surgical) during the acute phase, while intervention was performed in the chronic phase in six cases with PAUs’ growth. Over the follow-up period, in one case there was an evolution to AD, and a MAD increase >5 mm/2 weeks occurred in two patients (5.4%).

**Table 1:** Baseline characteristics of studies on penetrating aortic ulcers (PAU). Type B includes locations in the descending and abdominal aorta. All imaging markers that have been tested in order to identify an association with aortic-related adverse events are listed in the “radiologic features” column. MAD: maximum aortic diameter.

Study	Year	N° of PAUs (%)	N° of Type B (%)	Acute (n°)	Follow-Up	Radiologic Features
Jànosi et al. [14]	2016	63 (100)	52 (82.5)	63	45.6 months	PAU length, depth, neck width, longitudinal extension, MAD.
Yang et al. [11]	2020	109 (100)	76 (70%)	109	23 months	PAU length, depth, neck width, longitudinal extension, MAD, thickness of periaortic hematoma.
Squizzato et al. [5]	2022	28 (21)	28 (100)	9	8.1 years	PAU length, depth, neck width, longitudinal extension, MAD.
Piazza et al. [10]	2022	37 (68.5)	37 (100)	37	34 months	PAU length, depth, neck width, longitudinal extension, MAD, thickness of periaortic hematoma.

#### 3.2.2. IMH

ARM timing was specified in five studies [10,18,19,21,24], and it occurred in the acute phase in 17/433 cases (3.9%). The number of patients undergoing intervention was reported in 12 studies [10,12,13,16,17,18,20,21,22,23,24,25], with a rate of 21% during follow-up (241/1147 cases) (Table 2).

The outcome AAE was defined as composite of endovascular or surgical repair, aortic rupture, development of AD, progressive aortic enlargement and disease evolution, such as ULP (or FID) development [12,13,17,19,20,21,23,25]. The cumulative AAE rate based on seven studies was 32% (249/768 examined cases). According to Ishizu et al. [19], AAE onset was more frequent in patients with large FID at the time of diagnosis (15/28, 54%); meanwhile, according to Chen et al. [17], acute adverse events only happened in the subgroup of patients with obstructive sleep apnea (OSA).

Squizzato et al. [5] reported an estimated freedom from clinical disease progression at 1, 5 and 10 years of 44.4%, while the 10 years ARM estimated rate was 28.9%. The MAD had an increasing trend, with a grow-rate of 0.2 mm/year (IQR −0.1–0.6).

Bolomey et al. [15] divided their cohort in two groups depending on disease evolution. Cases showing an unfavorable disease progression (12/25, 48%) were characterized by an increase in MAD, and 11 of these underwent intervention.

**Table 2:** Baseline characteristics of studies on intramural hematomas (IMHs). Type B includes locations in the descending and abdominal aorta. All imaging markers that have been tested in order to identify an association with aortic-related adverse events are listed in the “radiologic features” column. MAD: maximum aortic diameter; MHT: maximum hematoma thickness; ULP: ulcer-like projections; FID: focal intimal disruptions; IBP: intramural blood pools; SUV_max_: maximum standardized uptake of the aortic wall; SUV_svc_: maximum standardized uptake of the superior vena cava; TBR (the SUV_max_/SUV_svc_): target to blood ratio.

Study	Year	N° of IMHs (%)	Type B (%)	Acute (n°)	Follow-Up	Radiologic Features
Sebastià et al. [22]	2012	34 (100)	34 (100)	34	5.9 years	MAD; MAD indexed with body surface; MHT; IMH length; ulcers; mediastinal hematoma.
Sueyoshi et al. [12]	2017	65 (100)	65 (100)	65	28 months	MAD, MHT, IBP, intimal defect, pleural fluid.
Sailer et al. [23]	2017	44 (32)	44 (100)	44	861 days	MAD.
Moral et al. [24]	2017	107 (100)	107 (100)	107	56 months	FID: presence, location, depth, orifice; MAD; MHT.
Liu et al. [21]	2018	123 (100)	84 (69)	116	20 months	MAD; MHT; atherosclerosis plaque; echo-lucent areas; circular shape.
Yang et al. [13]	2019	34 (100)	34 (100)	34	42 days	Initial ULP and MAD; Changes in ULP and MAD; SUVmax; SUVsvc; TBR.
Li et al. [25]	2019	238 (100)	238 (100)	238	543 days	IMH presence and location; ULP; IBP; MHT; MAD.
Bolomey et al. [15]	2020	25 (100)	25 (100)	25	18 months	IMH location and longitudinal extension, MHT, volume of IMH, MAD, aortic volume, diameter and volume of circulating lumen.
Chen et al. [16]	2020	226 (100)	226 (100)	226	45.9 months	MAD, MHT, aortic tortuosity, FID
Chen et al. [18]	2021	149 (100)	149 (100)	149	38.5 months	MAD, MHT, FID.
Ishizu et al. [19]	2021	76 (100)	76 (100)	76	4 years	MAD, MHT, Presence and location of FID, MHT at FID, calcified plaques adjacent to FID.
Yang et al. [20]	2021	61 (100)	61 (100)	61	167.3 weeks	MAD, MHT, MHT > 10 mm, ULP.
Squizzato et al. [5]	2022	12 (9)	11 (91.7)	8	8.1 years	IMH location and longitudinal extension, MHT, MAD.
Chen et al. [17]	2022	127 (100)	127 (100)	127	43 months	MAD, MHT, FID.
Piazza et al. [10]	2022	17 (31.5)	17 (100)	17	34 months	IMH location and longitudinal extension; MHT, ULP; IBP; MAD.

### 3.3. Markers of Disease Progression

#### 3.3.1. PAU

All articles identified imaging markers of disease progression. On multivariate analysis, the only factor related to disease progression, according to Squizzato et al. [5], was a PAU depth >10 mm, while smaller PAU width, neck and longitudinal extent were associated to disease resolution. Janosi et al. [14] detected a connection between mortality and a PAU depth >15 mm, while a PAU diameter and depth, respectively, of >12.5 mm and 9.5 mm were related to AAE according to the multivariate analysis of Yang et al. [11]. On univariate analysis, Piazza et al. [10] identified a PAU width >20 mm, PAU depth >15 mm, PAU depth/MAD >0.3 and disease located in the para-visceral aorta as markers related to the need of intervention in the acute phase, while in a chronic phase only MAD >35 mm was identified (Table 3).

**Table 3:** Focus on studies reporting on penetrating aortic ulcers (PAU). MAD: maximum aortic diameter; d: days; TEVAR: thoracic endovascular aortic repair; AD: aortic dissection; AAE: aortic-related adverse events.

Study	Year	Disease Progression	Aortic-Related Mortality	Radiologic Evolution	Predictors of AAE
Jànosi et al. [14]	2016	TEVAR < 14 d = 33/63 (52.3%); TEVAR 40 d = 30/63 (47.7%)	30 d = 5/63 (7.9%); 1 y, 5 y and 10 y survival = 88.4%, 84.2%, 65.7%	-	PAU depth > 15 mm
Yang et al. [11]	2020	30 d PAU AAE = 31/109 (28%); 30 d AD = 7/109 (6.4%); 30 d clinically-related adverse events = 7/109 (6.4%); Late aortic-related events = 34/109 (31%); Late AD 8/109 (7.3%); Late clinically related adverse events = 13/109 (12%)	30 d = 3/109 (2.7%); Late = 5/109 (4.6%)	Increase in PAU diameter and depth = 23/109 (21%)	PAU diameter > 12.5 PAU depth > 9.5 mm
Squizzato et al. [5]	2022	Freedom from clinical progression at 1, 5 and 10 y = 90.5%, 70.6% and 45.8%; 10 y PAU rupture rate = 7.2%; Disease resolution at 1, 5 and 10 y = 0%, 10.6%, 10.6%	10 y PAU mortality rate= 8.4%;	MAD increase = 0.4 mm/y (IQR 0–1.8); PAU depth increase = 0.5 mm/y (IQR 0.1–1.4); PAU width increase = 1.1 mm/y (IQR 0.7–1.7); Neck width increase = 1.1 mm/y (IQR 0.6–4.6)	PAU depth > 10 mm
Piazza et al. [10]	2022	Evolution to AD = 1/37 (2.7); TEVAR = 10/37 (27); Open 1/37 (2.7)	30 d = 1/37 (2.7)	MAD increase = 5 mm/2 weeks = 2/37 (5.4)	PAU width > 20 mm PAU depth > 15 mm PAU depth/total aortic diameter > 0.3 Paravisceral aortic PAU MAD > 35 mm

#### 3.3.2. IMH

Clinical and laboratory markers of disease progression and/or poor outcome were outlined in five studies [15,16,17,18,20], while in all articles the authors identified imaging predictors (Table 4).

According to Bolomey et al. [15], factors of poor prognosis were male gender, MAD > 38 mm and circulating lumen diameter >32 mm at the time of disease onset and MAD > 40 mm and circulating lumen diameter >34 mm after one month of PAU appearance. A higher C-reactive protein (CRP) level was connected to poor prognosis according to Chen et al. in two reports [16,18], together with FID development and an higher dosage of matrix-metalloproteinase-9 (MMP-9) [18]. Chen et al. [18] divided their cohort of patients into two groups depending on diabetes mellitus (DM): the group of patients without DM experienced more TEVARs in the acute phase, and the time lapse between disease onset and progression was shorter than patients without DM (6.4 vs. 12 days). The absence of DM was a factor related to ARM on univariate analysis (HR 3.82, 95% CI 1.57–9.28), although it did not reach statistical significance on multivariate analysis. In another report from Chen et al. [17], an higher mean morning systolic pressure influenced both ARM and AAE, while the presence of OSA was the only factor connected to ARM. According to Yang et al., an estimated glomerular filtration rate (eGFR) < 90 mL/min/1.73 m^2^ was connected to AAE, along with ULP development.

The presence of ulcers associated with IMHs was linked to AAE in 10 studies [10,15,16,18,19,20,21,22,24,25], although only seven of them made a distinction between PAUs and ULP or FID [10,16,18,19,20,24,25]. Piazza et al. [10] identified ULP and IMH extension > three aortic zones as factors related to the risk for intervention [10]. MAD was a marker of AAE in five articles [5,15,19,21]. In particular, a larger initial maximum aortic diameter (HR 1.18, 95% CI 10.02–1.32; *p* = 0.037) and IMH location in the aortic arch (HR 6.84, 95% CI 1.10–43; *p* = 0.040) were significantly associated with disease progression according to Squizzato et al. [5], while an initial MAD > 38 mm and a MAD > 40 mm after one month from diagnosis were linked to poor prognosis according to Bolomey et al. [15]. In addition, Ishizu et al. [19] identified a MAD ≥ 40 mm as predictor of AAE at multivariate analysis (HR 4.8; 95% CI: 1.8, 12.6; *p* = 0.001) along with larger FID diameter (hazard ratio, 3.2; 95% CI: 1.1, 8.9; *p* = 0.03). Minimum MAD related to AAE rose to 44.75 mm in the report of Liu et al. [21], and decreased to 38.2 mm according to Li et al. [25]. This last author [25] also identified changes in MHT (HR = 1.22, *p* < 0.001), ULP development (HR = 4.44, *p* < 0.001) and pleural effusion development (HR = 2.46, *p* = 0.002) as independently associated to AAE.

Finally, Yang et al. [13] analyzed 18F-FDG PET/CT of 34 patients with a type B IMH, identifying a TBR > 1.5 in patients with ULP as the only factor related to AAE.

**Table 4:** Focus on studies reporting on intramural hematoma (IMH). MAD: maximum aortic diameter; d: days; y: years; TEVAR: thoracic endovascular aortic repair; AD: aortic dissection; AAE: aortic-related adverse events; MHT: maximum hematoma thickness; ULP: ulcer-like projections; FID: focal intimal disruptions; TBR (the SUV_max_/ SUV_svc_): target to blood ratio; OSA: obstructive sleep apnea; DM: diabetes mellitus; FEG: favorable evolution group; UEG: unfavorable evolution group.

Study	Year	Clinical Progression	Aortic-Related Mortality	Radiologic Evolution	Predictors of AAE
Sebastià et al. [22]	2012	TEVAR = 2/34 (5.8)	0	Regression = 19/34 (56); Progression 15/34 (44); Regression of mediastinal hematoma = 4/4 (100); 7/10 ulcers progressed to aneurysm	Ulcers
Sueyoshi et al. [12]	2017	AAE = 23/65 (35%); Surgical repair = 7/65 (10.7)	3/65 (4.6)	Enlargement of ULP = 15/65 (24%)	EFL at initial CTA
Sailer et al. [23]	2017	Adverse events = 9/44 (21); AD = 7/44 (16); Resolution = 26/44 (59); TEVAR = 5/44 (11); 1 y, 2 y and 5 y probability of event-free survival = 76.5%, 76.5%, 68.9%	-	Non-resorbing IMH = Aortic grow-rate 1.49 mm/month	Non-reabsorption of IMH
Moral et al. [24]	2017	Early TEVAR = 10/107 (10); Late TEVAR = 7/94	30 d = 4/107 (4); Late = 1/94 (1)	FID development = 43/107 (40%) → 11 in the acute phase	FID development in the acute phase;
Liu et al. [21]	2018	TEVAR = 10/81 (12.3); 30 d AAE = 42/123; 30 d-6 m AAE = 7/81	30 d = 3/84 (3.5)	IMH regression = 68/123	Baseline MAD ≥ 44.75; Acute PAU development;
Yang et al. [13]	2019	AAE = 18/34 (53); TEVAR = 3/34 (8.8)	0	-	TBR > 1.5 in patients with ULP
Li et al. [25]	2019	AAE = 83/238 (40); Treatment = 76/238 (32);	Overall = 7/238 (3)	Median MHT and MAD change during follow-up = −1.5 mm and −1.1 mm; Newly developed ULP and IBP = 16 and 75	Baseline MHT ≥ 18.8; baseline MAD ≥ 38.2; ULP development; Changes in MHT; Pleural effusion development
Bolomey et al. [15]	2020	FEG = 13/25 pts (52%); UEG = 12/25 pts (48); 11 interventions;	2/25 pts (8%)	UEG: increase in aortic diameter	Male gender; Typical pain; IMH occurrence < 2015; Aortic diameter > 38 mm at D0 and 40 mm at M1; Circulating lumen diameter > 32 mm at D0 and 34 mm at M1; PAU at M1
Chen et al. [16]	2020	TEVAR= 27/187 pts (14.4%)	1 y: 28/187 (15%); postoperative: 13/27 (48%)	FID development = 61/187 (32.6); MHT increase = 6/187 (3.2)	FID development; Higher CRP level
Chen et al. [18]	2021	Acute phase TEVAR = DM 1/60 (2%); non-DM 11/89 (12%); Disease progression after onset = DM 12 d; non-DM 6.4 d	30 d = DM 0/60 (0%), non-DM 8/89 (9%); Late = DM 1/60(2%), non-DM 9/81 (11%)	Acute ULP = DM 0/60 (0%); non-DM 7/89 (7%); Acute signs of aortic rupture = DM 0/60 (0%); non-DM 8/89 (9%); Late worsening of hematoma = DM 3/60 (5%); non-DM 18/81 (22%)	ULP development during acute phase; Higher CRP level; Higher MMP-9 level
Ishizu et al. [19]	2021	AAE = no-FID 4 (15%); small-FID 2 (9%); large-FID 15 (54%);	30 d = 0; Late= no-FID 2/26 (8%), small-FID 1/22 (5%), large-FID 5/28 (18%);	Significant enlargement of large-FID	Large FID; MAD ≥ 40 mm
Yang et al. [20]	2021	AAE = 36/61 (59%); intervention = 24/61 (39%)	1/61 (1.6)	IMH progression 14/61 (23%); ULP progression 11/61 (18%)	ULP; eGFR < 90
Squizzato et al. [5]	2022	Freedom from clinical progression at 1, 5 and 10 y = 44.4%; The 10 y aortic rupture rate = 20%; Disease resolution at 1 and 5 y: 0%, 22.2%	10 y = 28.9 %	Aortic grow-rate = 0.2 mm/y (IQR −0.1–0.6)	Initial MAD; IMH location in the aortic arch
Chen et al. [17]	2022	Acute AAE = 17/76 (22%); Late TEVAR/surgery/reintervention = OSA 28/76 (37%); 2/51 (4%)	OSA = 9/76 (11.8%); non-OSA = 1/51 (1.9%)	Acute ULP = OSA 13/76 (17%); non-OSA 0/51 (0%); Late ULP = OSA 28/76 (37%); non-OSA 1/51 (2%); Late IMH resolution = OSA 41/76 (54%); non-OSA 49/51 (96%)	TEVAR; Higher mean morning systolic pressure; OSA;
Piazza et al. [10]	2022	Progression to AD = 2/17 (11.8); TEVAR =8/17 (47); Open = 3/17 (37.6)	Acute = 2/17 (11.8)	MAD > 5 mm/2 w = 4/17 (23.6)	ULP; IMH extension > 3 aortic zones

## 4. Discussion

PAUs and IMHs have always been considered as overlapping diseases [26]. However, recent literature [3,4,5,6] seems to suggest that differences in clinical course and outcomes could lead to two separated entities, although they can coexist and one can evolve into the other [1].

While PAUs primarily affect the aortic intima with a severe degree of atherosclerosis [27,28], IMHs rise in the aortic media. PAUs are rarely localized in the ascending aorta, and the disease progression description has always been controversial in the past literature; in fact, while some authors [27] reported a malignant course with a rupture rate of 40%, maybe because the ulcer involve more aortic layers, others successfully treated all patients with medical therapy [29]. The natural history of IMHs natural history is equally difficult to predict, since the progression rate to AD ranges from 28 to 47% [30].

In fact, because PAUs and IMHs have a consistently lower incidence compared to ADs, literature often describes natural history, prognosis factors and treatment modalities of all AASs, or mainly concentrates on ADs. Reports often merge type A and B localization and acute and chronic onset.

Despite the fact that specific RCTs are lacking, and most articles are observational and retrospective, recent studies have started to focus on the different behavior of PAUs and IMHs by identifying factors that could be related to a worsened disease prognosis, although data are heterogeneous [31].

In this work, we aimed to systematically review all studies of the last 10 years that specifically analyzed clinical, laboratory and imaging factors related to disease progression and AAE in patients affected by acute PAUs or IMHs, in order to identify patients who could benefit from an early intervention even if the disease is not immediately complicated.

First of all, 81% and 92% of patients analyzed in PAU articles were affected by a type B disease with an acute onset, respectively. Patients with IMHs presented with a disease located in the descending and/or abdominal aorta in 97% of cases, with a 99% rate of acute onset.

ARM of PAUs in the first 30 days from the disease onset was 4.3%, which was in line with other reports [32,33,34,35]. Most authors considered the composite outcome AAE, comprehensive of disease progression and need for intervention. Since estimated freedom from AAE progressively decreased over time [5,10,11], PAUs should be considered as a continuously evolving pathology, despite an optimal medical therapy, possibly leading to MAD increase and development of saccular aneurysms [10]. The examined literature confirms previous reports on minimum PAU depth associated to AAE [2,36], as the identified value ranges from 9.5 mm to 15 mm [5,10,11,14], while PAU diameter ≥ 12.5 mm was associated with aortic-related events according to Yang et al. [11] (versus ≥ 20 mm in previous reports [2,36]). As for MAD, Piazza et al. [10] noticed that a value > 35 mm was the only factor related to the need for intervention in the chronic phase, while previous reports [37] had identified a cut-off of 42 mm. None of the selected PAUs studied in this review investigated clinical and laboratory markers of AAE.

Cumulative in-hospital and 30-days ARM of IMHs was 3.9%, and 21% of patients underwent intervention during follow-up. It is interesting to note that freedom from disease progression remained stable over the follow-up period after the first year according to some authors [5], suggesting that, despite the fact that a wide percentage of patients affected by IMHs may have a disease progression during the first period [30], the disease tends to remain stable in the long-term, different from PAUs which tend to continuously evolve [5]. In our analysis, the presence of ulcers has been identified in 10/15 studies as a negative predictor of AAE, although only seven of these reports have made a distinction between PAUs and ULP (or FID). In fact, these two pathological conditions are often confused, and only recent literature is starting to make a distinction between IMHs with ULP and PAUs with hematoma, basing the diagnosis mainly upon the presence of atherosclerosis. Since many reports still encompass both conditions in the same group, further analyses are needed to assess which one is more associated with AAE risk. Other imaging markers were a MAD cut-off value ranging from 38 mm to 44.75 mm, which is slightly lower compared to previous literature (≥45 mm [36]), MHT changes and the presence of pleural effusion. Yang et al. [13] introduced the added prognostic value of the 18F-FDG PET/CT, where a TBR ≥ 1.5 in patients with ULP was related to AAE, although these findings are anecdotal and need to be confirmed by further studies. Five studies also reported on clinical factors (patients affected by OSA [17], non-diabetic patients [18], higher morning arterial pressure [17]) and laboratory factors (higher CRP value [16,18], higher MMP-9 level [18] and eGFR < 90 mL/min/1.73 m [2,20]), yet, once again, further studies are needed to confirm these results.

The main limitation of this review is the observational and retrospective nature of included studies. Furthermore, there is a heterogeneity of data about outcomes, selected treatments and overall findings, although many authors seem to agree on imaging markers leading to disease progression. Finally, since our goal was to summarize updates of recent literature analyzing factors related to AAE, the number of included PAUs articles is restrained, and findings are basically consistent with previous reports. Conversely, this work highlights that research on IMHs is continuously evolving.

## 5. Conclusions

Despite the fact that RCTs on IMHs and PAUs are lacking and current evidence is based on observational studies, the recent literature seems to suggest that cases that are not immediately complicated, yet presenting with imaging, clinical and laboratory markers of disease progression, could benefit from an early intervention, therefore reducing aortic-related adverse events. Of course, further research is warranted in order to confirm these findings and establish a correct and safe management of these uncommon diseases.
